# A novel interpretable machine learning framework for predicting postpartum depression: a SHAP-based analysis of maternal and infant health indicators

**DOI:** 10.3389/fpsyt.2026.1888858

**Published:** 2026-07-08

**Authors:** Feng Lv, Shufang Li, Xiang Yuan, Yan Ma, Tingyang Huang, Jiaan Xie, Baoying Feng, Jianqiu Zheng, Jifeng Feng, Jianlan Mo

**Affiliations:** 1 Department of Anesthesiology, Maternity and Child Health Care of Guangxi Zhuang Autonomous Region, Nanning, China; 2 Guangxi Clinical Research Center for Anesthesiology, Maternity and Child Health Care of Guangxi Zhuang Autonomous Region, Nanning, China; 3 Guangxi Academy of Medical Sciences, The People’s Hospital of Guangxi Zhuang Autonomous Region, Nanning, China; 4 Affiliated Ruikang Clinical Medical College of Guangxi University of Chinese Medicine, Nanning, China; 5 Department of Scientific Research, Maternity and Child Health Care of Guangxi Zhuang Autonomous Region, Nanning, China

**Keywords:** explainable artificial intelligence, machine learning, obstetric factors, postpartum depression, random forest, SHAP analysis

## Abstract

**Background:**

Postpartum depression (PPD) affects nearly 20% of women globally. Conventional regression models often have limited predictive accuracy.

**Objective:**

This study aimed to create and test a machine learning model for predicting PPD using comprehensive infant and maternal health indicators.

**Methods:**

In this prospective study, 273 postpartum women were enrolled, and data on 44 demographic, obstetric, and clinical variables were collected. After 1:2 propensity-score matching, participants were divided (7:3) into training and validation sets. Feature selection was executed using the least absolute shrinkage and selection operator (LASSO) regression. Nine machine learning algorithms were compared, including random forest, gradient boosting, support vector machines, and logistic regression. The Area Under Curve (AUC), calibration, and decision-curve analyses were employed to determine the performance of the model. The Shapley Additive exPlanations (SHAP) was utilized to explore model interpretability.

**Results:**

LASSO regression identified four key predictors of PPD: unplanned mode of delivery, premature rupture of membranes, NRS pain score at 10 cm cervical dilation, and socioeconomic subclass. Among the nine models tested, the random forest model exhibited superior overall performance, achieving an AUC of 0.952 in the training set and 0.745 in the hold-out validation set. SHAP analysis revealed that unplanned delivery and high intrapartum pain were the strongest positive contributors to PPD risk, while higher socioeconomic status served as a protective factor.

**Conclusion:**

The interpretable random-forest model, which integrates explainable artificial intelligence with obstetric data, accurately predicted PPD six weeks postpartum. It provides a practical tool for individualized screening and early intervention. Future multicenter studies that incorporate biological and psychosocial markers are needed to improve generalizability and applicability.

## 1 Introduction

Postpartum depression (PPD) affects women after childbirth. A meta-analysis encompassing over 172,000 women from 80 countries estimates the global prevalence of PPD at 17.2% (95% CI: 16.0–18.5%) (1, 2). In high-income regions, prevalence averages around 15%, while in poorer countries, it can reach approximately 19.8% (1, 3). PPD extends its impact far beyond maternal suffering. It not only increases maternal morbidity and suicide risk but also adversely affects children’s behavioral, emotional, and cognitive development. PPD imposes substantial burdens on healthcare systems, economies, and family well-being (4, 5). Despite its severity and prevalence, fewer than half of the women affected by PPD receive adequate psychiatric or psychosocial treatment. Thus, improved risk stratification and scalable early-intervention interventions are needed.

PPD is driven by multiple biological, psychological, and social factors including anxiety, limited social support, exposure to stressful life events, and socioeconomic disadvantage (6, 7). Increasing evidence highlights the importance of the mother-infant dyad in postpartum mental health. Maternal factors, including age, parity, body mass index, obstetric complications, delivery mode, and recovery quality, interact with infant factors such as neonatal illness and breastfeeding challenges. Together, these factors contribute to an individual’s vulnerability to PPD (8, 9). These interrelated stressors can prolong recovery, disrupt bonding, and heighten emotional distress (9). Most studies tend to focus on maternal or infant factors in isolation or use single-center samples, which limits their ability to capture complex nonlinear relationships. Additionally, conventional regression models often struggle with high-dimensional data. Furthermore, objective clinical indicators—such as physiological parameters, obstetric history, and neonatal outcomes—are underutilized, despite their potential to improve risk prediction (10, 11).

In recent years, machine learning (ML) has emerged as a promising tool for enhancing risk prediction in both mental health and perinatal medicine (12, 13). ML methods can analyze large, heterogeneous datasets, effectively capturing complex, nonlinear relationships between various predictors and outcomes (12, 13). Algorithms like random forests have shown better predictive performance than traditional models across different clinical contexts. Yet, ML is frequently challenged owing to its “black-box” nature, as model (14) decision-making is opaque and difficult to interpret, limiting clinical adoption (15). Consequently, explainable artificial intelligence (XAI) techniques, particularly SHAP, have gained prominence (16). Based on game theory, SHAP offers a consistent method to measure how each feature influences individual predictions, providing insights for a single output (local) and across the entire model (global) (16). This dual capability not only fosters trust and enhances clinical decision-making but also aids in identifying novel risk factors and interactions that conventional analyses might miss.

Given these limitations, we developed an interpretable framework for predicting the risk of PPD by integrating routinely collected maternal and infant health indicators. The novelty of this framework lies in its application rather than a modification of existing algorithms. Specifically, it jointly incorporates intrapartum pain measured at different stages of cervical dilation, obstetric events, and socioeconomic information. Furthermore, the framework compares nine complementary machine learning algorithms using a common validation workflow and employs SHAP to translate the final model into clinically interpretable global and individual risk explanations. This comprehensive approach is designed to support screening for PPD at six weeks postpartum using information readily available during routine perinatal care.

## 2 Methods

### 2.1 Study population

The study population consisted of pregnant women planning to undergo vaginal delivery at the Maternal and Child Health Hospital of Guangxi Zhuang Autonomous Region between September 2023 and February 2025. Participants were excluded based on the following criteria (1): a history of mental disorders; (2) presence of severe underlying diseases; (3) inability to provide informed consent due to intellectual or behavioral impairments; (4) confirmed or suspected prior drug abuse or alcohol; or (5) other circumstances deemed by the investigators to hinder participation or complicate follow-up, such as frequent changes in the work environment that could lead to potential loss to follow-up.

This study enrolled 273 eligible participants. Postpartum depression (PPD) symptoms were evaluated six weeks after delivery via the Edinburgh Postnatal Depression Scale (EPDS). Postnatal Depression Scale (EPDS), with a score of ≥13 indicating the presence of PPD. The study was approved by the Ethics Committee of the Maternal and Child Health Hospital of Guangxi Zhuang Autonomous Region (Ethics Approval No. [2025-7-1]) and registered in the Chinese Clinical Trial Registry (Registration No. ChiCTR2100044067).

### 2.2 Data collection

Patient information was gathered utilizing a standardized case report form created by the research team, which was adapted from established clinical guidelines and existing case records. The form encompassed extensive data pertaining to participants’ demographic characteristics, obstetric and medical histories, physical status, vital signs, and pertinent laboratory and clinical parameters. Overall, 273 women who satisfied the inclusion and exclusion criteria were enrolled in this study. PPD was assessed at 6 weeks postpartum using the EPDS, a validated 10-item self-reported questionnaire (17). Items are scored from 0 to 3. The sum of all items (0–30) indicates the level of depressive symptoms, with higher scores representing greater severity (17). In line with conventional thresholds and previous validation studies, an EPDS score of ≥ 13 was used to define PPD, while a score of< 13 indicated the absence of clinically significant depressive symptoms.

Before analysis, data completeness, plausible ranges, and coding consistency were verified against the case-report forms. The matched modeling dataset contained complete information for both outcomes and candidate predictors, precluding the need for statistical imputation. To improve future multicenter adoption, the preprocessing pipeline should prespecify missingness indicators and multiple imputation within each resampling fold to prevent information leakage.

### 2.3 Statistical analysis and feature selection

All categorical variables were summarized as frequencies and percentages. Initially, forty-four demographic and perinatal characteristics were included as candidate predictors. Following propensity score matching (PSM: caliper=0.025, ratio=1:2, based on education), a total of 129 participants were included. Subsequent analysis proceeded in three stages: 1) data partitioning into training (70%) and validation (30%) sets; 2) model development on the training set using LASSO regression with tenfold cross-validation for feature selection and parameter tuning; and 3) hold-out validation of the final model’s predictive performance. To ensure model parsimony, the optimal penalty parameter λ-1se (the λ value corresponding to the minimum partial likelihood deviance minus one standard error) was adopted for constructing the most parsimonious model.

Feature-selection stability was addressed conservatively through tenfold cross-validation and the λ-1se rule. However, selection frequencies across repeated resamples were not estimated in the present analysis. Recommended extensions are bootstrap stability selection (the complete selection procedure is repeated in bootstrap samples, and variables are retained according to prespecified selection-frequency thresholds) and elastic-net sensitivity analysis (to assess whether correlated predictors excluded by LASSO carry reproducible information).

Performance was assessed in the training set and independently evaluated in the hold-out validation set. Based on the optimal features derived from LASSO, nine machine learning models were constructed: K-Nearest Neighbor (KNN), Support Vector Machine (SVM), Logistic Regression, Gradient Boosting Machine (GBM), Neural Network, Random Forest (RF), Extreme Gradient Boosting (XGBoost), Adaptive Boosting (AdaBoost), and Light Gradient Boosting Machine (LightGBM) (18). These models were selected beforehand to embody different learning assumptions. Logistic regression served as an interpretable linear benchmark; KNN represented distance-based learning, while SVM evaluated margin-based classification. A neural network was included for its flexible nonlinear mapping; RF assessed bagged decision trees and their interactions. GBM, XGBoost, AdaBoost, and LightGBM each represented distinct boosting strategies. By comparing these diverse model families using the identical data partition and feature set, the study minimized reliance on any single modeling assumption. The quantitative metrics used for evaluation included accuracy (ACC), precision, sensitivity, specificity, and F1 score, where TP, TN, FP, and FN denote true positive, true negative, false positive, and false negative, respectively. In addition, model performance was further assessed through the receiver operating characteristic (ROC) curve, calibration curve, and decision curve analysis (DCA). Additionally, model interpretability was examined using Shapley Additive Explanations (SHAP) to visualize both global and individual feature contributions to the risk of PPD in the optimal model.

All statistical analyses were conducted using R version 4.3.3 and SAS 9.4, with a two-tailed P ≤ 0.05 considered statistically significant.

The sample size for this study was determined by the existing prospective cohort rather than through an *a priori* ML-specific power calculation. With 129 matched participants and 43 PPD events, the dataset presents limitations for evaluating 44 initial candidate predictors and nine algorithms. Current recommendations suggest tailoring prediction-model sample size to factors such as outcome prevalence, candidate predictor parameters, and expected model fit. These guidelines generally advocate for utilizing all available data with bootstrap internal validation over a single data split (19). Consequently, this analysis is presented as a proof-of-concept. Future validation efforts will incorporate a prespecified sample-size calculation, repeated nested cross-validation or bootstrapping, and an independent external cohort.

### 2.4 Mathematical formulations and model evaluation

For binary PPD outcome *y_i*∈{0,1} and predictor vector *x_i*, LASSO logistic regression estimated coefficients by minimizing:


$$\hat{\beta }=\text{arg}\underset{\beta }{\min}\left\{-\frac{1}{n}\sum_{i}\left[y_{i}\log(p_{i})+\left(1-y_{i}\right)\log(1-p_{i})\right]+\lambda \sum_{j}\left|\beta_{j}\right|\right\},$$

where $p_{i}=\frac{1}{1+exp\left[-\left(\beta_{0}+x_{i}^{T}\beta \right)\right]}$.

The L1 penalty, by shrinking weak coefficients to zero, makes LASSO particularly well-suited for parsimonious feature selection, especially when the number of candidate predictors is substantial relative to the training sample size (20). The *λ-1se* rule was used to favor a stable, less complex subset.

The candidate classifiers represented different functional forms. Logistic regression used $p_{i}=\frac{1}{1+exp\left[-\left(\beta_{0}+x_{i}^{T}\beta \right)\right]}$. KNN assigned the majority class among the *k* nearest observations. SVM estimated a maximum-margin boundary by minimizing $\frac{1}{2}{\left\|\text{w}\right\|}^{2}+C\sum_{i}\xi_{i}$ subject to $y_{i}\left({\text{w}}^{T}\phi \left(x_{i}\right)+b\right)\geq 1-\xi_{i}$. A neural network estimated $\hat{y}=\sigma \left({\text{W}}_{2}g\left({\text{W}}_{1}x+b_{1}\right)+b_{2}\right)$. RF averaged B bootstrapped decision trees, ${\hat{p}}_{RF}\left(x\right)=B^{-1}\sum_{b}T_{b}\left(x\right)$ (21). Boosting models formed an additive predictor $F_{M}\left(x\right)=\sum_{m}\eta_{m}h_{m}\left(x\right)$, with implementation-specific loss functions and regularization.

Model discrimination and classification were evaluated using AUC and the following threshold-based metrics: Accuracy = (TP + TN)/(TP + TN + FP + FN), Sensitivity = TP/(TP + FN), Specificity = TN/(TN + FP), Precision = TP/(TP + FP), and F1 score = 2 × Precision × Sensitivity/(Precision + Sensitivity). The AUC represents the probability that a randomly selected participant with PPD will have a higher predicted risk than a participant without PPD. Calibration curves were used to assess the agreement between predicted and observed risk.

Clinical utility was assessed by decision-curve net benefit: $NB=\frac{TP}{n}-\frac{FP}{n}\times \frac{p_{t}}{1-p_{t}}$, across threshold probability *p_t* (22). SHAP represented each prediction as $f\left(x\right)=\phi_{0}+\sum_{j}\phi_{j}$, where *φ_j* is the Shapley contribution of feature *j* (16). Given that LASSO coefficients are penalized and SHAP values elucidate predictions rather than causal effects, the retained variables were construed as predictive contributors. Consequently, conventional P values were not employed to assert post-selection statistical significance.

## 3 Results

### 3.1 Participant characteristics

This study enrolled 273 participants, with a mean age of 30.66 ± 4.31 years (details are presented in **Table 1**). After applying 1:2 propensity score matching, 129 participants were selected for further analysis. These participants were then split into a training set (n=92) and a test set (n=37). Additional information on the baseline characteristics and delivery-related parameters of the participants can be found in **Table 2**.

**Table 1:** The baseline characteristics of our study.

Variables	Total (n = 273)	0 (n = 230)	1 (n = 43)	Statistic	*P*
Age, Mean ± SD	30.66 ± 4.31	30.72 ± 4.28	30.37 ± 4.50	t=0.48	0.631
Gravidity, Mean ± SD	2.26 ± 1.31	2.27 ± 1.30	2.26 ± 1.38	t=0.04	0.966
Weight-gain-during-pregnancy, Mean ± SD	13.85 ± 5.28	13.97 ± 5.18	13.19 ± 5.80	t=0.89	0.372
BMI, Mean ± SD	26.95 ± 3.22	26.89 ± 3.24	27.27 ± 3.13	t=-0.71	0.479
NRS-pain-score-At0-cm-cervical-dilation, Mean ± SD	7.10 ± 2.78	7.00 ± 2.81	7.63 ± 2.56	t=-1.37	0.171
NRS-pain-score-At3-cm-cervical-dilation, Mean ± SD	4.47 ± 2.87	4.39 ± 2.90	4.88 ± 2.73	t=-1.03	0.303
NRS-pain-score-At10-cm-cervical-dilation, Mean ± SD	6.90 ± 3.20	6.66 ± 3.25	8.19 ± 2.58	t=-3.42	**0.001**
Duration-of-NA, Mean ± SD	7.02 ± 6.34	6.87 ± 6.32	7.85 ± 6.51	t=-0.93	0.353
Duration-of-labor-First-stage, Mean ± SD	459.21 ± 292.51	452.07 ± 292.75	497.40 ± 291.64	t=-0.93	0.352
Duration-of-labor-Second-stage, Mean ± SD	48.59 ± 42.76	48.50 ± 43.63	49.09 ± 38.22	t=-0.08	0.934
Hospital-stays, Mean ± SD	3.12 ± 1.17	3.12 ± 1.19	3.12 ± 1.10	t=0.01	0.995
Duration-of-pregnancy, Mean ± SD	275.64 ± 6.60	275.80 ± 6.48	274.79 ± 7.22	t=0.92	0.36
Birth-weight, Mean ± SD	3199.85 ± 354.94	3198.35 ± 355.01	3207.91 ± 358.65	t=-0.16	0.872
Family-income, n (%)				χ²=2.67	0.446
1	79 (28.94)	67 (29.13)	12 (27.91)		
2	100 (36.63)	83 (36.09)	17 (39.53)		
3	70 (25.64)	62 (26.96)	8 (18.60)		
4	24 (8.79)	18 (7.83)	6 (13.95)		
Education, n (%)				–	0.795
1	22 (8.06)	18 (7.83)	4 (9.30)		
2	191 (69.96)	160 (69.57)	31 (72.09)		
3	36 (13.19)	30 (13.04)	6 (13.95)		
4	24 (8.79)	22 (9.57)	2 (4.65)		
Premenstrual-syndrome-before-pregnancy, n (%)				χ²=1.50	0.221
0	144 (52.75)	125 (54.35)	19 (44.19)		
1	129 (47.25)	105 (45.65)	24 (55.81)		
Disfavorable-pregnancy, n (%)				χ²=0.00	1
0	262 (95.97)	221 (96.09)	41 (95.35)		
1	11 (4.03)	9 (3.91)	2 (4.65)		
Discomfort-in-early-pregnancy, n (%)				χ²=3.92	0.048
0	93 (34.07)	84 (36.52)	9 (20.93)		
1	180 (65.93)	146 (63.48)	34 (79.07)		
Discomfort-in-late-term-pregnancy, n (%)				χ²=5.26	0.022
0	70 (25.64)	65 (28.26)	5 (11.63)		
1	203 (74.36)	165 (71.74)	38 (88.37)		
Medication-during-pregnancy, n (%)				χ²=0.64	0.425
0	233 (85.35)	198 (86.09)	35 (81.40)		
1	40 (14.65)	32 (13.91)	8 (18.60)		

t, t-test; χ², Chi-square test; -, Fisher exact; SD, standard deviation. The bold values indicate statistically significant differences (P < 0.05).

**Table 2:** The baseline characteristics of propensity score matching.

Variables	Total (n = 129)	0 (n = 86)	1 (n = 43)	Statistic	*P*
Age, Mean ± SD	30.56 ± 3.82	30.65 ± 3.45	30.37 ± 4.50	t=0.36	0.722
Gravidity, Mean ± SD	2.16 ± 1.25	2.12 ± 1.19	2.26 ± 1.38	t=-0.59	0.554
Weight gain during pregnancy, Mean ± SD	13.54 ± 5.83	13.71 ± 5.87	13.19 ± 5.80	t=0.48	0.632
BMI, Mean ± SD	27.10 ± 3.13	27.01 ± 3.15	27.27 ± 3.13	t=-0.44	0.66
NRS pain score At0 cm cervical dilation, Mean ± SD	7.39 ± 2.63	7.27 ± 2.67	7.63 ± 2.56	t=-0.73	0.466
NRS pain score At3 cm cervical dilation, Mean ± SD	4.54 ± 2.80	4.37 ± 2.84	4.88 ± 2.73	t=-0.98	0.33
NRS pain score At10 cm cervical dilation, Mean ± SD	6.97 ± 3.19	6.36 ± 3.31	8.19 ± 2.58	t=-3.44	**<.001**
Duration of NA, Mean ± SD	7.47 ± 6.35	7.27 ± 6.30	7.85 ± 6.51	t=-0.48	0.629
Duration of labor First stage, Mean ± SD	485.29 ± 290.31	479.23 ± 291.16	497.40 ± 291.64	t=-0.33	0.739
Duration of labor Second stage, Mean ± SD	48.59 ± 42.11	48.34 ± 44.13	49.09 ± 38.22	t=-0.10	0.924
Hospital stays, Mean ± SD	3.18 ± 1.22	3.21 ± 1.28	3.12 ± 1.10	t=0.41	0.685
Duration of pregnancy, Mean ± SD	275.72 ± 6.63	276.19 ± 6.30	274.79 ± 7.22	t=1.13	0.261
Birth weight, Mean ± SD	3200.54 ± 356.33	3196.86 ± 357.21	3207.91 ± 358.65	t=-0.17	0.869
Subclass, Mean ± SD	29.00 ± 14.22	32.50 ± 13.76	22.00 ± 12.56	t=4.20	**<.001**
Family income, n (%)				χ²=2.84	0.418
1	31 (24.03)	19 (22.09)	12 (27.91)		
2	51 (39.53)	34 (39.53)	17 (39.53)		
3	34 (26.36)	26 (30.23)	8 (18.60)		
4	13 (10.08)	7 (8.14)	6 (13.95)		
Education, n (%)				–	1
1	12 (9.30)	8 (9.30)	4 (9.30)		
2	93 (72.09)	62 (72.09)	31 (72.09)		
3	18 (13.95)	12 (13.95)	6 (13.95)		
4	6 (4.65)	4 (4.65)	2 (4.65)		
Premenstrual syndrome before pregnancy, n (%)				χ²=0.76	0.383
0	64 (49.61)	45 (52.33)	19 (44.19)		
1	65 (50.39)	41 (47.67)	24 (55.81)		
Disfavorable pregnancy, n (%)				χ²=0.02	0.897
0	121 (93.80)	80 (93.02)	41 (95.35)		
1	8 (6.20)	6 (6.98)	2 (4.65)		
Discomfort in early pregnancy, n (%)				χ²=0.98	0.323
0	34 (26.36)	25 (29.07)	9 (20.93)		
1	95 (73.64)	61 (70.93)	34 (79.07)		
Discomfort in late term pregnancy, n (%)				χ²=3.37	0.066
0	27 (20.93)	22 (25.58)	5 (11.63)		
1	102 (79.07)	64 (74.42)	38 (88.37)		
Medication during pregnancy, n (%)				χ²=0.11	0.741
0	107 (82.95)	72 (83.72)	35 (81.40)		
1	22 (17.05)	14 (16.28)	8 (18.60)		

t, t-test; χ², Chi-square test; -, Fisher exact. The bold values indicate statistically significant differences (P < 0.05).

### 3.2 Feature selection

**Figure 1:**
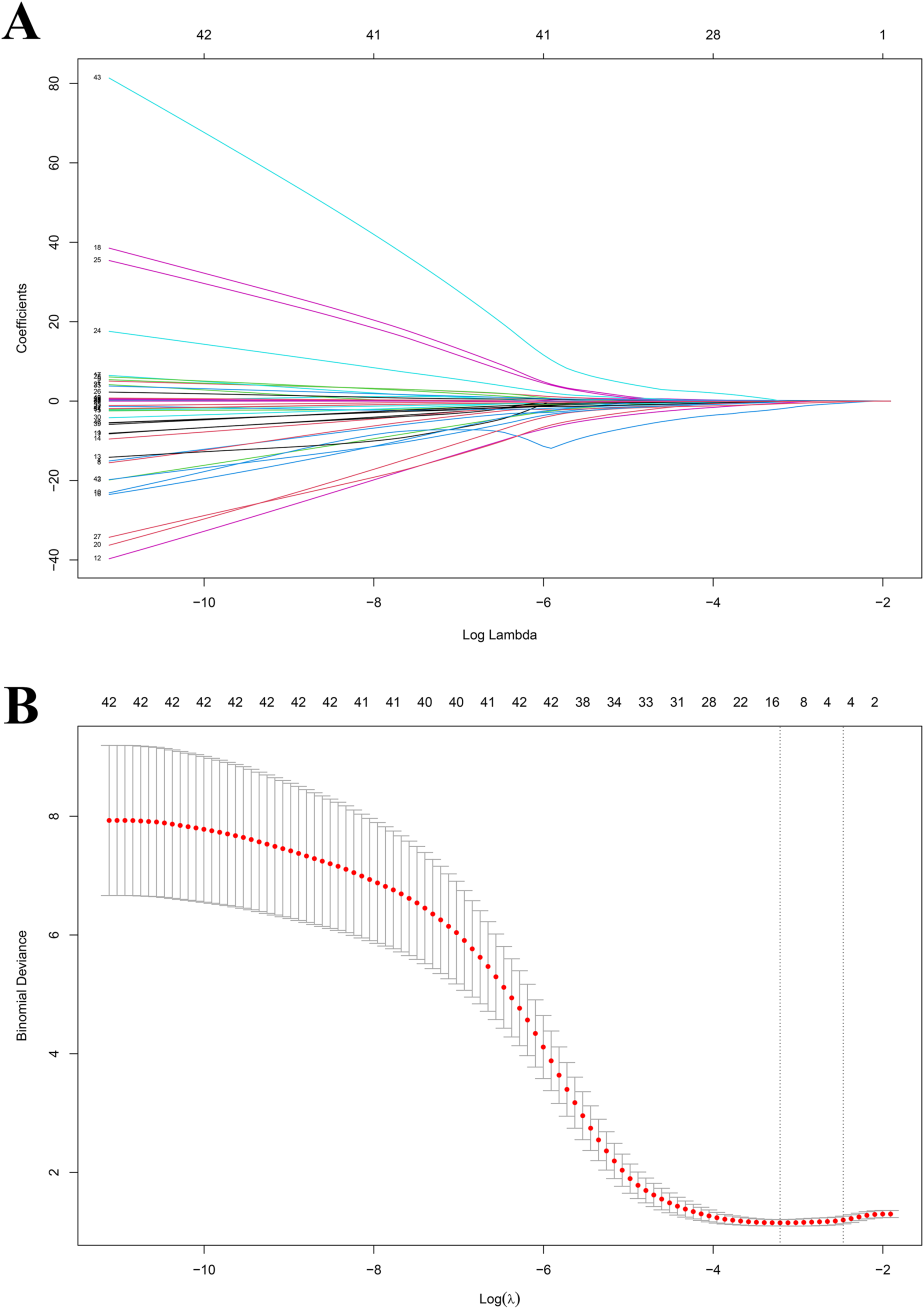
**(A)** Coefficient path plot of Lasso regression versus log(λ). **(B)** Binomial Deviance plot of Lasso regression versus log(λ).

A total of 44 potential risk factors associated with postpartum depression were initially considered, including basic demographic and intrapartum characteristics. To reduce the number of variables and identify the most representative features, LASSO regression was applied to the training set. The optimal penalty parameter (λ) was determined using 10-fold cross-validation. For model parsimony, the final model was selected based on the one standard error (1se) rule corresponding to the optimal λ value, which also determined the number of variables retained with non-zero regression coefficients. The Lasso regression analysis identified four significant variables potentially influencing postpartum depression: unplanned mode of delivery, premature rupture of membranes, NRS pain score at 10 cm cervical dilation, and subclass (**Figure 1**).

### 3.3 Evaluation and comparison of machine learning models

**Figure 2:**
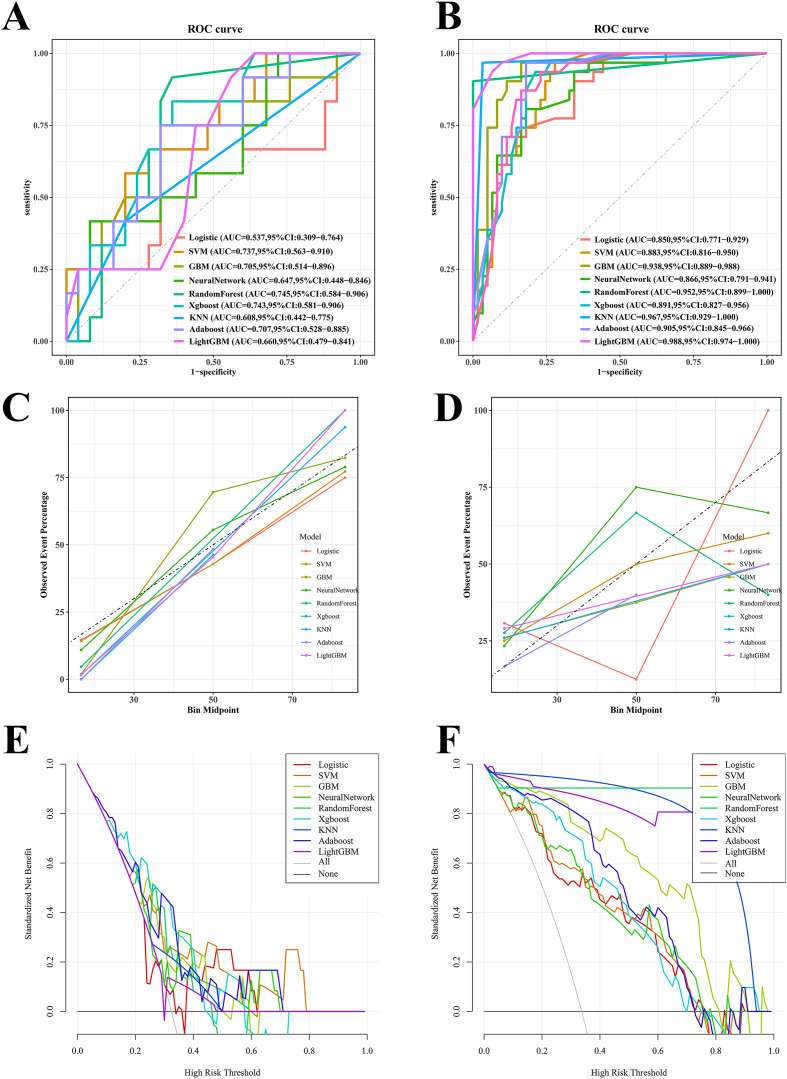
**(A)** ROC curves of the nine machine learning models on the validation set. **(B)** ROC curves of the nine machine learning models on the training set. **(C)** Calibration Curves for the machine learning models in the training set. **(D)** Calibration curves for the machine learning models in the validation set. **(E)** Decision curve analysis of the machine learning models in the training set. **(F)** Decision curve analysis (DCA) of the machine learning models in the validation set.

The four potential factors for postpartum depression identified by the Lasso regression were used to develop nine machine learning algorithms: LightGBM, AdaBoost, KNN, XGBoost, Random Forest, Neural Network, GBM, SVM, and Logistic Regression. A comprehensive comparison of their performance was conducted, revealing that the Random Forest risk prediction model outperformed the others in the validation set. Specifically, it achieved an AUC of 0.745 (**Figure 2A**), an accuracy of 0.73, a sensitivity of 0.917, a specificity of 0.64, a precision of 0.55, and an F1-score of 0.687. Based on these findings, the Random Forest model was selected as the final model (**Table 3**).

**Table 3:** Performance metrics of the machine learning models on the validation set.

Model	Threshold	Accuracy	Sensitivity	Specificity	Precision	F1
NA	NA	NA	NA	NA	NA	NA
Logistic	0.66	0.76	0.25	1.00	1.00	0.40
SVM	0.19	0.70	0.67	0.72	0.53	0.59
GBM	0.06	0.70	0.83	0.64	0.53	0.65
NeuralNetwork	0.32	0.76	0.42	0.92	0.71	0.53
RandomForest	0.00	0.73	0.92	0.64	0.55	0.69
Xgboost	0.35	0.70	0.83	0.64	0.53	0.65
KNN	0.50	0.68	0.42	0.80	0.50	0.46
Adaboost	0.39	0.70	0.75	0.68	0.53	0.62
LightGBM	0.10	0.57	1.00	0.36	0.43	0.60

### 3.4 ROC curves, calibration curves, and DCA curves

Among the nine constructed machine learning models, the AUC (95% CI) values in the training set were as follows: SVM scored 0.883 (95% CI: 0.816-0.950), Neural Network scored 0.866 (95% CI: 0.791-0.941), and Random Forest achieved the highest score of 0.952 (95% CI: 0.899-1.000) (**Figure 2B**). In the test set, the corresponding AUC (95% CI) values were 0.737 (95% CI: 0.563-0.910) for SVM, 0.647 (95% CI: 0.448-0.846) for Neural Network, and 0.745 (95% CI: 0.584-0.906) for Random Forest (**Figures 2A, B**). The calibration curves for the different machine learning algorithms showed that the curve for the Random Forest algorithm closely approximated the diagonal in both the training and test sets. This indicates that the model’s predicted probabilities aligned closely with the actual observed probabilities, demonstrating a high level of consistency with the ideal prediction curve (**Figures 2C, D**).

Across a threshold probability range of 0 to 0.65, the Random Forest model demonstrated a positive net benefit in DCA. Its model-based strategy consistently yielded a superior net clinical benefit compared to the default “treat-all” and “treat-none” strategies, highlighting its practical value for clinical decision-making within this interval (**Figures 2E, F**).

The DCA range should not be interpreted as a single validated treatment threshold. For implementation, a lower threshold will prioritize sensitivity for low-burden actions such as repeat EPDS screening or referral for psychosocial assessment, whereas a higher threshold could be reserved for resource-intensive specialist evaluation. The operating threshold must be prospectively selected in collaboration with clinicians and patients. This selection should consider the local prevalence of PPD, the capacity for referrals, and the respective implications of false-negative and false-positive alerts. Following this selection, the threshold should be evaluated in a clinical-impact study.

### 3.5 Confusion matrix

**Figure 3:**
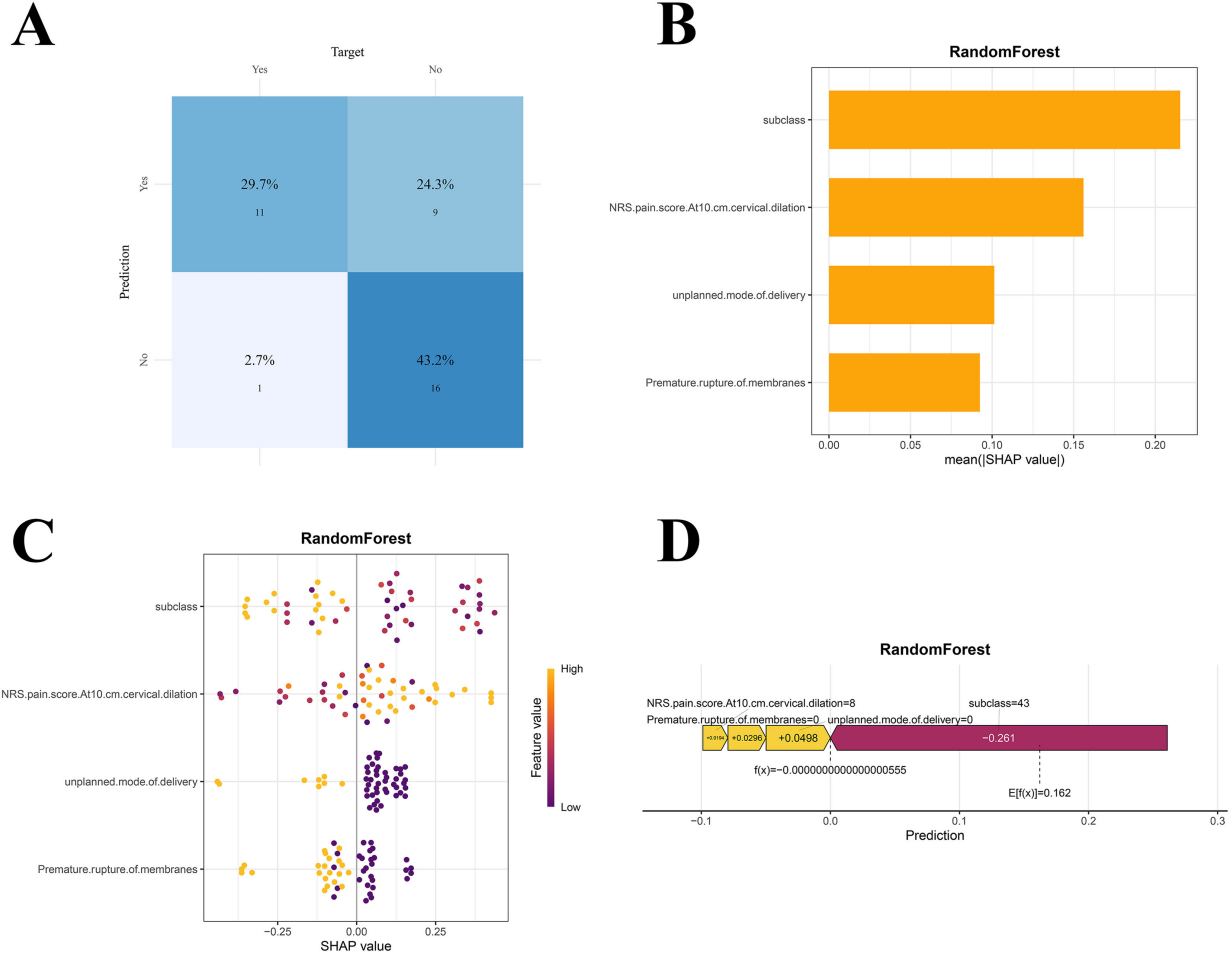
**(A)** Confusion matrix of the random forest model on the validation set. **(B)** Bar plot of random forest feature importance. **(C)** SHAP beeswarm plot for the random forest model. **(D)** SHAP force plot explaining an individual prediction from the random forest model.

Evaluation using the confusion matrices revealed highly consistent performance metrics for both the training and test sets. This consistency suggests potential for generalizability, though the small hold-out validation set prevents a definitive claim of robustness (**Figure 3A**). Specifically, the test set achieved the following performance metrics: an accuracy of 0.73, sensitivity of 0.917, specificity of 0.64, precision of 0.55, and an F1-score of 0.687.

### 3.6 Interpretation of the random forest model based on SHAP analysis

This study employed SHAP (SHapley Additive exPlanations) for global and local interpretation of the Random Forest model. Positive SHAP values indicate a shift in prediction toward the PPD class relative to the model’s baseline output, while negative values indicate a shift toward the non-PPD class. SHAP magnitude indicates the strength of contribution, not causality or statistical significance. For categorical predictors, directionality is contingent upon the specified numerical coding; therefore, text interpretations were validated against beeswarm colors, feature coding, and individual force plot.

The SHAP analysis revealed the feature importance ranking, indicating that the unplanned mode of delivery held the strongest predictive value for postpartum depression, followed by premature rupture of membranes, NRS pain score at 10 cm cervical dilation, and subclass, which also demonstrated significant predictive importance (**Figure 3B**).

LASSO was employed for feature selection, not as the primary predictive model. Its appropriateness stemmed from the dataset’s structure: 44 potential predictors were available for only 92 training observations after matching and splitting, which posed a considerable risk of overfitting. The L1 penalty and the λ-1se rule yielded a sparse, clinically practical feature set prior to model comparison. However, LASSO might select only one variable from a set of correlated predictors and may overlook nonlinear or interaction-only signals. Consequently, alternative or hybrid pipelines—such as elastic net, stability selection, recursive feature elimination, Boruta or random-forest importance, and stacked ensembles—could potentially retain additional predictors. In this modest sample, evaluating numerous feature-selection/model combinations without nested cross-validation would inflate optimism and introduce selection bias; such methodologies warrant assessment in larger multicenter cohorts using nested resampling. Recent explainable-ML studies on PPD similarly indicate that the chosen predictors and the optimal algorithm differ based on the population, timing, and available variables (23–25).

Thus, this proposed re-evaluation of the current LASSO pipeline aims not to supplant LASSO with a more intricate method in this limited dataset, but rather to assess its reproducibility. Future research should involve comparing the original LASSO subset with elastic net and bootstrap stability selection within a nested cross-validation framework. This work should report selection frequencies and performance optimism, while only retaining additional predictors if they demonstrably enhance externally validated discrimination, calibration, or clinical utility.

The distribution of each feature’s effects on the model output is visualized in the beeswarm plot (**Figure 3C**), illustrating the positive or negative relationships between the predictors and the likelihood of postpartum depression. Each point in the plot represents the SHAP value for a single patient-instance: purple reflects a lower feature value, while yellow denotes a higher feature value. **Figure 3C** illustrates that a higher NRS pain score at 10 cm cervical dilation and an unplanned delivery positively correlated with an increased predicted risk of PPD. Conversely, a higher socioeconomic subclass generally predicted a lower risk. Given that the direction of categorical SHAP effects is contingent on variable coding, these observed patterns were interpreted in conjunction with the feature definitions and individual-level explanations.

The SHAP force plot for the Random Forest model, displayed for a specific instance, was used to explain the prediction for an individual sample. For a patient who did not develop postpartum depression, the primary contributing factors identified were a value of ‘43’ for the `subclass` feature and an NRS pain score at 10 cm cervical dilation of ‘0’. This waterfall plot clearly illustrates the strength of each feature’s contribution to the final prediction outcome for this specific instance. It highlights the key drivers behind the model’s decision for this individual prediction (**Figure 3D**).

## 4 Discussion

This study developed and internally evaluated an interpretable ML model to predict PPD at six weeks postpartum. By integrating demographic and perinatal variables, we evaluated nine ML algorithms and identified the Random Forest model as the most effective predictor. This model exhibited superior discrimination and calibration performance, while SHAP analysis offered clear explanations for individual predictions. Our findings indicate that explainable ML methods could serve as a practical tool for the early identification of women at risk for PPD in real-world clinical settings.

The novelty of this study lies in its clinically focused integration and application, rather than the invention or mathematical modification of LASSO, RF, or SHAP. Compared to previous PPD prediction studies, our workflow prioritizes routinely collected, delivery-proximal variables, such as pain at full cervical dilation and unplanned delivery. It also links parsimonious variable selection, broad algorithm comparison, hold-out validation, SHAP interpretation, calibration, and decision-curve analysis. This comprehensive approach creates an interpretable connection between intrapartum observations and individualized six-week PPD screening. The framework adheres to contemporary guidelines for transparent clinical prediction-model studies (26).

Previous research has shown that PPD is a multifactorial condition influenced by obstetric, psychological, and socioeconomic determinants. While traditional regression-based methods have been employed to identify associated risk factors, their predictive power is limited due to reliance on linear assumptions, which fail to account for the complex, nonlinear interactions inherent in perinatal data (27). In recent years, ML methods have demonstrated a superior ability to model complex phenomena. For instance, a large cohort study utilizing gradient boosting identified antenatal anxiety, sleep disturbance, and delivery complications as the primary predictors of PPD, achieving an AUC of 0.82 (28). Interpretable ML models have also been utilized in psychiatric research to predict depression relapse and suicide risk. These models demonstrate that factors such as social isolation, inflammatory markers, and hormonal changes significantly enhance prediction accuracy when compared to conventional models (29, 30). Building on this evidence, our Random Forest model exhibited strong discriminative performance and high interpretability through SHAP analysis. This confirms that transparent AI-based tools can enhance clinical understanding and individualized risk assessment in maternal mental health.

LASSO regression identified four key variables associated with PPD among the 44 candidate predictors: unplanned mode of delivery, premature rupture of membranes, NRS pain score at 10 cm cervical dilation, and subclass. The Random Forest model, which incorporated these features, achieved the highest predictive accuracy and generalization performance. SHAP interpretation further revealed that unplanned delivery had the strongest influence on PPD risk, supporting the hypothesis that unexpected or emergent obstetric experiences may increase maternal psychological stress (31). Similarly, premature rupture of membranes, which is often linked to prolonged labor and increased perinatal anxiety, contributed to a higher risk of PPD (32). Higher intrapartum pain intensity at full cervical dilation was positively correlated with depressive symptoms. This finding aligns with evidence indicating that uncontrolled pain may activate stress-related neuroendocrine pathways (23). The “subclass” variable, which represents socioeconomic position, was found to be inversely related to PPD risk. This suggests that lower social support or limited resources may increase vulnerability to postpartum mood disorders (23). Together, these findings underscore the interactive role of both physiological and psychosocial factors in shaping postpartum mental health outcomes.

The mechanisms connecting obstetric and psychosocial factors to PPD are likely multifaceted, involving complex biological and behavioral pathways. Obstetric complications, such as unplanned delivery and premature rupture of membranes, can initiate increased inflammatory and neuroendocrine responses. This includes the activation of the hypothalamic–pituitary–adrenal (HPA) axis and dysregulation of cortisol secretion, both of which have been associated with depressive symptoms (33, 34). Intense intrapartum pain at full cervical dilation can exacerbate stress-related hormonal fluctuations, which may result in neuroplastic changes in brain regions responsible for mood regulation (35). Concurrently, the psychosocial consequences of complicated deliveries— including prolonged recovery, functional limitations, and impaired mother–infant bonding—may exacerbate emotional distress (36, 37). Socioeconomic disadvantage can increase vulnerability by restricting access to social and healthcare support, which in turn intensifies feelings of isolation and inadequacy (38). These convergent pathways indicate that PPD does not stem from a single precipitating event. Instead, it results from a cumulative interplay of physiological and psychosocial dysregulation (39). Understanding these mechanisms highlights the importance of integrating biological markers and behavioral data into explainable machine learning frameworks. This integration can clarify causal pathways and inform targeted preventive strategies.

The interpretable Random Forest model developed in this study represents a promising digital tool for individualized screening and prevention of PPD. Compared to conventional regression-based scores, it achieved higher AUC, sensitivity, and specificity, facilitating more reliable identification of high-risk women. SHAP analysis offered visual explanations of how specific factors contributed to risk, thereby enhancing clinical confidence and improving patient communication. Once externally validated, the model could be integrated into electronic health records to generate automated alerts and support timely psychological interventions. By combining advanced machine learning techniques, rigorous feature selection, and propensity score matching, we minimized confounding factors and bolstered model robustness. Initial model performance was evaluated through training and hold-out validation. SHAP analysis enhanced transparency by providing clinically interpretable predictions, thereby mitigating the “black-box” nature of the model. However, independent multicenter validation is still essential.

A practical implementation strategy involves integrating the four routinely available predictors into the electronic health record post-delivery. This allows for risk calculation before discharge or during postpartum follow-up. Displaying the primary SHAP contributors and linking the alert to a repeat EPDS assessment and a defined referral pathway would further enhance its utility. Prior to deployment, the model requires extensive validation, including multicenter temporal and geographic validation, recalibration in regions with different baseline PPD (postpartum depression) prevalence, assessment of subgroup fairness, workflow usability testing, prospective impact evaluation, and continuous monitoring for performance drift.

Several limitations must be acknowledged. First, the effective sample size was modest (n=129; n=92 for training; n=37 for validation) and originated from a single geographic region. The validation AUC of 0.745, significantly lower than the training AUC of 0.952 suggests potential optimism and uncertainty. Consequently, this study should be viewed as a proof-of-concept rather than a deployment-ready model. Future research necessitates larger multicenter prospective cohorts, repeated or nested cross-validation, external validation, and formal sample-size planning, in accordance with current prediction-model guidelines (26). Second, PSM (propensity score matching) reduced the available sample and may have led to information loss. Future investigations should compare matching with weighting or covariate adjustment techniques. Third, while LASSO promotes sparsity, it can be unstable with correlated predictors and does not directly capture nonlinear or interaction-only effects. Alternative methods such as elastic net, stability selection, embedded tree-based selection, and hybrid or ensemble pipelines could identify additional predictors, but these approaches require nested resampling to avoid selection bias. Fourth, the study primarily incorporated clinical and demographic variables. Integrating validated psychosocial scales, psychiatric history, social support, hormonal profiles, and inflammatory markers could improve prediction and better reflect the established multifactorial etiology of PPD. Fifth, SHAP explains model predictions but does not establish causality or conventional statistical significance, and its values can fluctuate among correlated features. Finally, before implementation, there is a need for temporal models, clinical-impact studies, fairness assessments across socioeconomic groups, cost-effectiveness analyses, and prospective workflow evaluations.

## 5 Conclusions

This study constructed a novel application-oriented, interpretable Random Forest framework for predicting postpartum depression six weeks following delivery. The integration of explainable machine learning techniques, specifically SHAP analysis, yielded high predictive performance alongside clear interpretability, thereby bridging the divide between computational modeling and clinical application. By elucidating the collective influence of obstetric factors, intrapartum pain, and socioeconomic status on PPD risk, this research underscores the potential of interpretable AI to enhance personalized mental health screening and early intervention for postpartum women. Future integration of such models into clinical and community health systems may facilitate proactive, data-driven strategies aimed at improving maternal mental health on a global scale.

## Data Availability

The original contributions presented in the study are included in the article/supplementary material. Further inquiries can be directed to the corresponding authors.
